# Flexible letter-position coding in Chinese-English L2 bilinguals: Evidence from eye movements

**DOI:** 10.1177/17470218241229442

**Published:** 2024-02-28

**Authors:** Hillarie Man, Adam J Parker, J. S. H. Taylor

**Affiliations:** Division of Psychology and Language Sciences, University College London, London, UK

**Keywords:** transposed-letter, letter-position coding, second-language processing, bilingualism, eye movements, sentence reading

## Abstract

Theories suggest that efficient recognition of English words depends on flexible letter-position coding, demonstrated by the fact that transposed-letter primes (e.g., JUGDE-judge) facilitate written word recognition more than substituted-letter primes (e.g., JUFBE-judge). The multiple route model predicts that reading experience should drive more flexible letter-position coding as readers transition from decoding words letter-by-letter to recognising words as wholes. This study therefore examined whether letter-position is coded flexibly in second-language English sentence reading for native Chinese speakers, and if this is influenced by English proficiency. Eye movements were measured while 54 adult native Chinese speakers read English sentences including either a real word (e.g., cheaply), a transposed-letter nonword (e.g., “*chepaly*”), or a substituted-letter nonword (e.g., “*chegely*”). Flexible letter-position coding was observed in initial and later processing stages—reading times were longer for substituted-letter than transposed-letter nonwords. In addition, reading times were longer in both initial and later processing stages for transposed-letter nonwords than real words, indicating that, despite encoding letter-position flexibly, readers processed letter-position. Although pre-registered frequentist analyses suggested that English proficiency did not predict overall reading times, Bayes Factors indicated that there was evidence for such a relationship. It is therefore likely that this proficiency analysis suffered from low power. Finally, neither frequentist nor Bayes Factor analyses suggested that English proficiency influenced the difference in reading times between different target word types, i.e., the nature of letter-position coding. Overall, these results suggest that highly proficient L2 learners code letter-position flexibly.

The fact that readers of alphabetic orthographies can distinguish between anagrams (e.g., *pirates—parties)* indicates that we must encode the order in which letters occur within words. However, the well-replicated transposed-letter (TL) effect, whereby nonwords created by transposing two letters of a word (e.g., table—talbe) are perceived as more like their base words than substituted-letter (SL) nonwords (e.g., table—tarpe), indicates that readers encode letter-position with a degree of flexibility (Grainger, 2018). Such flexible letter-position coding is embodied in the direct orthographic-to-semantic route of the multiple route model of printed word recognition and enables efficient mapping of word forms onto meanings (Grainger et al., 2012). In contrast, the phonologically mediated route of this model encodes letters serially and maps them onto individual phonemes. This leads to the prediction that reading experience should result in more flexible letter-position coding as readers transition from decoding words letter-by-letter to recognising words as wholes (Grainger et al., 2012; Share, 1995; Ziegler et al., 2014). Adult language learners offer an opportunity to study the influence of reading experience on letter-position coding, independent of maturational confounds. Capitalising on this, the current study was a partial replication of Cong and Chen (2022), and examined reading times for target words, TL nonwords, and SL nonwords in Chinese-English bilinguals’ reading of English sentences. We expected that both types of nonword would show longer reading times than target words, and that TL nonwords would show shorter reading times than SL nonwords. We further expected that proficiency would increase the TL relative to SL nonword advantage.

## Models of visual word recognition

It is well-replicated that lexical decisions to target words are faster when preceded by a TL masked prime formed by swapping two letters of a target word, than by an SL prime formed by replacing the same letters (e.g., *caniso* vs. *carivo* for the target word *CASINO;* Perea & Lupker, 2004; Stinchcombe et al., 2012). This TL effect (TL faster than SL primed words) is greater when at least one of the TLs is a consonant, when the distance between the TLs is small, and when the transposition includes only inner letters (Johnson et al., 2007; Ktori et al., 2014; Perea et al., 2008; Perea & Lupker, 2003, 2004). Early computational models of visual word recognition, such as the Interactive Activation (IA) model (McClelland & Rumelhart, 1981), do not capture these patterns of behaviour, because they encode letter order using position-specific (or slot-based) coding, in which each letter is represented by a separate bank of letter units.

More recent models of visual word recognition can account for TL effects. As described in Lupker et al. (2019), one-class of models is noisy position models, such as the spatial coding model (Davis, 2010), the Bayesian reader (Norris, 2006), and the overlap model (Gomez et al., 2008). These models all assume that letter position is encoded with a degree of uncertainty. For example, for a letter in position 4, there is some probability that it occurs in positions 3 or 5, or to a lesser extent positions 1 or 6. Noisy position coding accounts for the perceived similarity between TL primes and target words since there is some probability that these letters are in the same position in the two items. A second type of model is the Letters in Time and Retinotopic Space (LTRS) model (Adelman, 2011). The main assumption of the LTRS is that information about letter identity and letter order accumulates over time, but that this information will ultimately be encoded correctly. This model accounts for TL effects because, at a point in time at which letter identity and position information are uncertain, a TL prime may have a similar representation to a target word. In contrast, any identification of the replaced letters in an SL prime will signal that this is not the same as the target word. A final class of models are those that use open-bigrams to encode relative letter position. Letter identity and order are represented as a bag of bigrams—letter pairs that are ordered but not necessarily adjacent. For example, TABLE would be coded as TA, TB, TL, TE, AB, AL, AE, BL, BE, and LE. Overlap between these open-bigrams accounts for TL and other forms of relative position priming (e.g., tbl priming TABLE; Grainger et al., 2006; Whitney et al., 2012).

**Figure 1.**
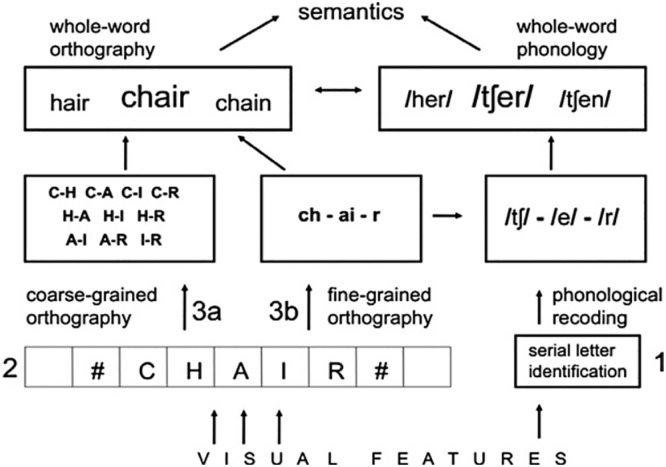
Multiple route model of reading, taken from Grainger et al. (2012).

Noisy position models and the LTRS simulate many phenomena pertaining to visual word recognition, such as the degree of facilitation that occurs with primes in which target word letters are replaced, transposed, deleted, or inserted. However, they do not incorporate mechanisms for mapping from orthographic to phonological or semantic information. Our study was therefore situated in the context of the multiple route model (Figure 1; Grainger et al., 2012), which comprises three-routes to understanding a written word, one of which incorporates open-bigram coding.

One route of the multiple route model involves phonological recoding prior to semantic access (right-hand side of Figure 1). Letters (or letter combinations/graphemes) are identified and mapped onto individual phonemes before a word’s phonological representation is accessed, followed by its meaning. Phonological recoding requires serial letter identification (fine-grained orthographic processing) such that the letters in a word are processed from left to right so that they can be mapped onto their corresponding sounds in the correct order. A second route also uses fine-grained orthographic processing but maps directly from orthography to semantics (middle section of Figure 1). Grainger et al. (2012) suggested that this might be particularly important for extracting combinations of letters that form affixes, which have a consistent relationship between letters and meaning (e.g., ED signals the past tense, UN signals an opposite). The third route is the primary mechanism for mapping orthography directly onto semantics (left-hand side of Figure 1). Letters are encoded in parallel rather than serially and in a coarse-grained, or flexible, manner in the form of open-bigram coding. Grainger et al. (2012) proposed that coarse-grained orthographic processing maximises the amount of information available regarding word identity, enabling efficient word identification and access to meaning.

## The role of reading experience

Children learning alphabetic orthographies first learn to read words by sounding them out letter-by-letter, using taught knowledge of how letters correspond to sounds. Once they have sounded out a word, they can use their spoken vocabulary knowledge to understand its meaning (Castles et al., 2018). In the multiple route model (Grainger et al., 2012), this corresponds to beginning readers primarily using the phonological recoding route to understand written words. With increasing reading experience, children begin to recognise written words as wholes and rapidly access their meanings, without such a need for phonological recoding (Castles et al., 2018; Share, 1995). This entails a gradual shift from phonological recording to direct print-to-meaning mapping, which also implies decreasing reliance on fine-grained, and increasing reliance on coarse-grained, orthographic processing (Grainger et al., 2012).

To examine the influence of reading experience/expertise on orthographic coding, we first consider studies that have compared rejection times for TL and SL nonwords in a lexical decision task. TL nonwords are typically harder to reject and Perea et al. (2005) found that this was more pronounced for TL nonwords created from high than low-frequency words, whereas SL nonwords were minimally affected by base word frequency. This suggests that TL nonwords activate base word representations in the direct print-to-meaning route. Supporting the idea that coarse-grained letter-position coding increases with reading experience and/or maturation, Grainger et al. (2012) found that TL nonwords were harder to reject than SL nonwords, and that this difference was greater for older children and adults than younger children. However, somewhat opposing effects were reported by Perea et al. (2016), who found that TL > SL nonword rejection times were reduced for expert scrabble players relative to typical university students. Gomez et al. (2021) also reported that the difference in rejection times for TL compared to SL nonwords was reduced for 11- to 12-year-olds who were better pseudoword readers. However, this relationship is difficult to explain since the multiple route model proposes that word (not pseudoword) reading ability should drive increasing use of coarse-rather than fine-grained coding. Furthermore, as discussed by Ziegler et al. (2014), complex decision operations are involved in making NO responses to nonwords in lexical decision tasks, and the inconsistent effects discussed in this paragraph cannot therefore be unequivocally attributed to maturational or item-level differences in orthographic representations.

Other studies have used the masked priming lexical decision task, which avoids this issue since the critical comparison is between YES responses to the same target word preceded by different primes. Lété and Fayol (2013) reported greater TL than SL priming for adults but equivalent priming for children. Providing evidence for a gradual increase in flexible letter-position coding with age/experience, Ziegler et al. (2014) found that the difference between TL and SL priming increased between the ages of 6 and 10. These findings support the view that flexible letter-position coding increases with reading experience and/or maturation. However, others have reported null effects of age on TL versus SL priming. Acha and Perea (2008) found that 7- and 11-year-olds and adults showed stronger TL than SL masked priming in a lexical decision task, with no difference in the magnitude of this difference between the groups. Similarly, neither Hasenäcker and Schroeder (2022) nor Kezilas et al. (2017) reported any age differences in TL relative to SL priming for children aged 7 to 10. Overall, the literature is somewhat mixed as to whether flexible letter-position coding increases with reading proficiency and/or maturation, as predicted by the multiple route model, with some negative findings (though these have typically not used optimal methods to examine this particular question), some null effects, and some positive findings.

## Adult language learners

Adult language learners offer an opportunity to study the influence of reading experience on letter-position coding, independent of maturational confounds. Perea et al. (2011) found that Spanish intermediate Arabic learners showed stronger masked priming from Arabic TL than SL nonwords. Lin and Lin (2016) used mouse-tracking technology to track hand-movements towards YES or NO options as participants decided whether a letter string was a real English word or not. Both Chinese-English and Spanish-English bilinguals displayed the TL effect, whereby they took longer to reject TL nonwords as real words compared to SL nonwords. The mouse trajectories also demonstrated that participants were more strongly pulled towards the “YES” response for TL than SL nonwords. These findings suggest that flexible letter-position coding is also present in L2 learners.

One issue to consider in studies of L2 orthographic processing is that L1 orthography and phonology may impact L2 processing, particularly if both use alphabetic writing systems. Wang et al. (2003) examined English reading in Korean L1 (alphabetic) and Chinese L1 (non-alphabetic) speakers. Korean speakers were more reliant on phonological than orthographic information in identifying English words, whereas the opposite was true for Chinese speakers. It may therefore be prudent to study bilinguals whose L1 and L2 use different orthographic systems when investigating the development of letter-position coding in non-native speakers. For example, as well as having many syntactic and grammatical differences from English (Choi & Gopnik, 1995; Gentner, 1982; Lee & Naigles, 2005), Chinese uses a logographic written system consisting of morphemic units with no spaces between characters. Chen et al. (2020) found that for native Chinese speakers, TL primes facilitated lexical decisions to English target words more than SL primes for both low and high proficiency L2 English learners. However, this TL effect was only present when target words were high frequency. This suggests that L2 learners of alphabetic orthographies do show flexible letter-position coding that is not dependent on a cross-over from their native language orthography, and that this is related to the development of whole-word orthographic representations.

## Eye-tracking to study sentence reading

Most studies investigating letter-position coding have used isolated word presentation, which does not reflect real reading. However, eye-tracking technology allows letter-position coding to be examined during *online* sentence reading. White et al. (2008) found that word-initial TLs generated longer reading times than TLs in the middle or end of a word, with larger effects for low than high frequency words. However, the effect was only evident for total reading time, a reading measure that encompasses both early and later stages of word processing. Pagán et al. (2021) similarly found that transposing the first and third letters caused more disruption in total reading time than internal transpositions for both children and adults. In addition, transposition effects emerged in earlier measures for adults than children and were more pronounced for more skilled than less skilled child readers. In adults, Blythe et al. (2014) found that fixation times on TL nonwords significantly increased when the distance between the TLs increased, and when the two TLs included a consonant and a vowel rather than two consonants or two vowels. Blythe et al. (2014) also found that meaningful sentence contexts facilitated processing and identification of TL words relative to isolated word presentation, highlighting the importance of studying flexible letter-position coding within more real-world contexts. These studies did not include SL nonwords as a comparison, which makes it difficult to isolate effects of letter-position from those of letter identity on reading times. This is yet to be examined in English L1 sentence reading using eye-tracking, though there is evidence that overall sentence reading times are longer when target words are replaced with SL than TL nonwords (Rayner & Kaiser, 1975).

## Cong and Chen (2022)

Cong and Chen (2022) used eye-tracking to investigate the flexibility of letter-position coding in Chinese university students’ English L2 sentence reading. Eye movements were recorded during single-sentence reading, with target words in each sentence presented in one of six conditions: two within-morpheme conditions (Within-TL, Within-SL), two between-morpheme conditions (Between TL, Between Between-TL, Between-SL), the identity (ID) condition (original target word), and a baseline non-word condition (formed by replacing two letters of the between-TL nonword while retaining the stem). TL nonwords for the between- and within-morpheme manipulations were created by switching two adjacent letters of the base word (ID) and SL nonwords were created by replacing the same letters with other letters. Between- and within-morpheme manipulations varied whether the swapped letter positions cross a morpheme boundary (e.g., *golefr* vs. *gofler* for the word *golfer*). Consonants were always switched for consonants and vowels for vowels. Four eye movement measures were examined: first fixation duration and gaze duration, to index early processing stages, and go past time and total reading time, to index late processing stages. They observed significantly shorter gaze durations, go past times, and total reading times in the ID condition than the Within-SL condition, and in the Within-SL condition compared to the Within-SL condition. This supports the existence of flexible letter-position coding in L2 orthographic representations and suggests that such codes are activated during both early and later stages of sentence processing. However, surprisingly, the Within-SL and ID conditions had equivalent reading times, suggesting that this flexibility may be even greater than in L1 readers. In the between-morpheme conditions, go past time and total reading time were significantly shorter in the Between-TL condition compared to the Between-SL condition, but this effect was not significant for gaze duration nor first fixation duration. Though findings for the between-morpheme conditions are harder to interpret, the within-morpheme conditions suggest that Chinese-English bilinguals demonstrate flexible letter-position coding in their L2 English sentence reading.

Cong and Chen’s (2022) observation of flexible letter-position coding in L2 English readers is important, yet their study raises several questions. First, reading times were much longer (>1,000 ms in total reading time) than those observed for native English speakers in other studies (Godfroid et al., 2018; Martin & Juffs, 2021; Mézière et al., 2023). These substantially longer reading times may indicate that participants’ overall proficiency in English could have moderated the pattern of effects. Therefore, it is imperative to explore this possibility in a separate study that not only focuses on TL effects but also how they interact with proficiency, particularly since the multiple route model predicts that proficiency influences letter-position coding. To examine the proficiency effects in a meaningful way, we decided to only use items from Cong and Chen’s within-morpheme condition so that we had 40 observations per participant for the ID condition, the TL condition, and SL condition, instead of 20 items if we had included the full set of stimuli. Given that the majority of research has focused on within-morpheme manipulations, and those looking at between-morpheme manipulations have yielded mixed findings (Cong & Chen, 2022; Kahraman & Kırkıcı, 2021; Perea et al., 2011b; Zeng et al., 2019), we feel that this was an advantageous decision. A second question is whether Cong and Chen’s study was sufficiently well-powered to detect reliable effects across eye movement measures. Given that Cong and Chen incorporated no formal power analysis into their study, it is difficult to judge whether any null effects were due to issues of power or an absence of an effect. Therefore, with slow, iterative, cumulative science in mind, we felt it worthwhile to attempt to both replicate Cong and Chen’s within-morpheme findings and examine proficiency effects.

## Aims and hypotheses

The current study aims to investigate the flexibility of letter-position coding in Chinese native speakers’ L2 English sentence reading, by replicating the within-morpheme condition from Cong and Chen (2022). We also aim to explore how L2 proficiency may influence letter-position coding by examining whether TL effects relate to English proficiency, as measured using the LexTALE English vocabulary test (Lemhöfer & Broersma, 2012). The multiple route model predicts that for L1 reading, increased exposure to and proficiency with a writing system will drive a transition from sequential fine-grained orthographic processing to parallel coarse-grained processing. More proficient readers should therefore show more flexible letter-position coding as indexed by a greater TL effect, i.e., a greater advantage in reading times for TL than SL nonwords. We pre-registered several predictions that applied to all eye-tracking measures (gaze durations, go past times, total reading time):

(1) Longer reading times for TL and SL nonwords compared to real word targets.
(2) Longer reading times for SL than TL nonwords.
(3) A main effect of proficiency, with longer reading times for less than more proficient readers.
(4) Higher relative to lower proficiency English speakers will show a bigger difference in reading times between
 (a) TL and SL nonwords compared to real word targets and
 (b) SL compared to TL nonwords.

As explained further in the *Method* we powered our study to replicate Cong and Chen‘s (2022) within-morpheme conditions. We did not, however, power our study to detect the interactions between transposition and proficiency. This was partly because we did not have robust evidence to obtain effect sizes from, and partly because a large sample size would be uneconomical should the LexTALE turn out to be a poor predictor of eye movement measures. We therefore consider our analyses of (1) and (2) as confirmatory and our analyses of (3) and (4) akin to an exploratory analysis in the knowledge that there is potential for this to be underpowered.

## Method

This study was pre-registered prior to the commencement of data collection. The pre-registration, data, and analysis code can be found on the OSF: https://osf.io/2va7x/. The pre-registration reports how we determined our sample size, data exclusions, manipulations, and measures in the study.

### Participants

Sixty-two Chinese-English bilingual speakers were recruited through University College London’s SONA Psychology Subject Pool. Participants were native speakers of Chinese and had experience speaking and reading English, and were aged 18–40 (see Data analysis for demographic information on our post-exclusion final sample). They had normal or corrected-to-normal vision and had no hearing impairments or a history of reading disorder. They were naïve to the purpose of the experiment and gave written informed consent prior to participation.

Ethical approval for this project was granted through UCL’s Experimental Psychology Department Ethics Chair (Project ID: EP/2021/015).

### Materials

We used 120 items from Cong and Chen (2022). We selected items with a within-morpheme manipulation where target words were manipulated so that sentences contained a TL, SL, or identical (ID) target.

ID words were real words (e.g., *cheaply*) and were 120 derivational words that ranged from 5 to 13 letters (*M* = 7.86), with two to four syllables. All items were morphologically complex, meaning that word length was relatively long. On a scale of one to five, with five being more familiar, the ID words were rated 4.27 on familiarity (*SD* = .54; range = 3.00–5.00) by 20 students in Cong and Chen (2022) with similar language proficiency to their formal participants. SUBTLEX-US was used to calculate the word frequency of ID words, which, on a log scale, had a mean of 2.41 (*SD* = .56, range = .48–3.39). ID words had a mean orthographic neighbourhood density of 2.30 (*SD* = .57, range = 1.45–4.20), which refers to the average orthographic distance from the nearest 20 orthographic neighbours, where lower scores suggest denser neighbourhoods.

TL nonwords were formed by switching two adjacent letters of each ID word (e.g., *chepaly* from *cheaply*) and SL nonwords were formed by replacing the switched-letters with other letters (e.g., *chegely*), with vowels always substituted for vowels and consonants for consonants. Cong and Chen (2022) also calculated summed bigram frequencies of the nonwords and reported that the transposed (*M* = 223,124, *SD* = 279,606) and substituted (*M* = 193,217, *SD* = 327,558) nonwords were not significantly different from each other, *t*(119) = 1.48, 95% CI = [–10,200.08, 70,013.91], *p* = .14.

**Figure 2.**
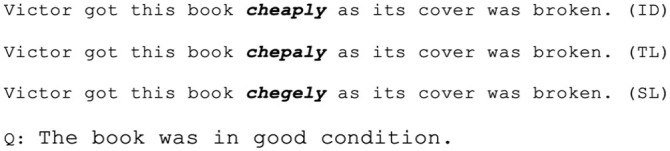
Example stimuli sentence and comprehension question where the target word is presented in ***bold*** for each condition. Note. Participants saw all text as regular Courier New text.

The target items were placed in 120 single-line sentences (see Online Appendix A https://osf.io/2va7x/files/), which ranged from six to 15 words long. Figure 2 shows an example sentence from each condition. Target words never appeared as the first two words or the last word in the sentence. There were 86 simple and 34 complex sentences, where simple sentences contained the subject, predicate, and object(s), and complex sentences also included a clause. The target real words’ probability (or their predictability from context) in each sentence was evaluated by Cong and Chen (2022). They asked 10 college or graduate students with similar language proficiency to their formal participants to guess the next word from the context preceding the target word. Only two items were corrected and guessed by two participants separately, suggesting that overall, target words were not predictable from the prior context.

Also shown in Figure 2, we created 40 comprehension statements based on a third of these sentences, where 20 were True and 20 were False (see Online Appendix C https://osf.io/2va7x/files/). These comprehension statements did not require participants to understand the meaning of the target word and were used to ensure that participants were paying attention and understood the overall meaning of the sentences.

Three counterbalanced lists of the experimental sentences were created, such that each target word appeared in each of the three conditions (ID, TL, and SL) across the lists (see Online Appendix B https://osf.io/2va7x/files/). Participants were assigned to one of these counterbalanced lists; thus, of the 120 target words, each participant was presented with 40 words for each condition, meaning 40 TL nonword sentences, 40 SL nonword sentences, and 40 ID word sentences.

The English LexTALE (Lemhöfer & Broersma, 2012) and Chinese LexTALE (Chan & Chang, 2018) were used in this study as measures of proficiency. The LexTALEs are short, un-speeded lexical decision tasks consisting of 60 trials (40 real words and 20 pseudowords) for English and 90 trials for Simplified Chinese (60 real words and 30 pseudowords) presented in a randomised order. We used the Gorilla Experiment Builder (www.gorilla.sc) to create and host our experiment (Anwyl-Irvine et al., 2020). Participants are tasked with deciding whether a string or character was a real word in the language or not by clicking “yes” or “no” buttons on the screen. Each trial started with a fixation cross for 250 ms, followed by the item. After the participant responds, a second fixation cross is displayed for 250 ms before the next trial begins. Scores are calculated as the average of the proportion of words correct and the proportion of pseudowords correct. The English LexTALE has been suggested to be a good predictor of English vocabulary knowledge, to correlate highly with general English proficiency measures, and to be superior to self-rated proficiency in its predictions (Lemhöfer & Broersma, 2012).

### Apparatus

Sentences were displayed in 20-point black *Courier New* on a Dell U2414H monitor with a 1,920 by 1,080 display. A headrest for head stabilisation was set up 87 cm from the screen, and the stimuli was displayed at 15 pixels wide per character such that each character took up 0.27 degrees of visual angle on the retina. While viewing was binocular, gaze position was sampled via an EyeLink Portable Duo at a rate of 1,000 Hz (i.e., once per millisecond) for the right eye for all but one participant whose left eye was recorded.

### Design

This experimental study included one within-subject manipulated factor of item type: TL vs. SL vs. identity (ID), and one continuous between-subject variable of English proficiency.

Gaze position was used to compute three eye movement measures: gaze duration (the sum of all first-pass fixations on a word before moving to another), go past time (the sum of the fixation durations on the target from the first fixation until the gaze falls to the area to the right of the target), and total reading time (the sum of all fixation durations on the target). These intercorrelated measures are indicative of both early and late stages of lexical processing (Liversedge et al., 1998).

### Statistical power

To ensure sufficient power, sample size for the partial replication of Cong and Chen (2022) was determined from power simulations following DeBruine and Barr (2021). First, the data structure (i.e., variables and factors such as item type) and the fixed effects and random effect parameters (i.e., grand mean and mean differences from Cong & Chen, 2022) were specified. This subsequently allowed the sampling of stimuli items, subjects, trials, and response values. Full details for the power simulations can be found on the OSF (https://osf.io/2va7x/files/).

Effect sizes were estimated by fitting linear mixed-effects models with Helmert contrasts to the within-morpheme data provided by Cong and Chen (2022). Contrasts were set such that comparison one compared TL and SL vs. ID, and comparison two compared TL vs. SL. These power calculations determined that 7 participants per counterbalanced list (i.e., a total of 21 participants) would be sufficient to partially replicate findings from Cong and Chen (2022) with at least ~90% power. Table 1 shows the power achieved for each dependent measure at this sample size at an alpha level of p< .017 (corrected for multiple comparisons of eye movement measures, i.e., .05/3).

**Table 1.** Power and effect size simulations for 21 participants for contrast 1 (TL and SL vs. ID) and contrast 2 (TL vs. SL) for each of the three measures: gaze duration, go past time, and total reading time.

Measure	Contrast	Power (%)	Effect size (log(ms) units)
**Gaze duration**	Contrast 1 (TL and SL vs ID)	90.6	–.030
	Contrast 2 (TL vs SL)	91.2	.059
**Go past time**	Contrast 1 (TL and SL vs ID)	100.0	–.050
	Contrast 2 (TL vs SL)	98.6	.069
**Total reading time**	Contrast 1 (TL and SL vs ID)	89.4	–.050
	Contrast 2 (TL vs SL)	100.0	.122

TL: transposed letter; SL: substituted letter; ID: identity.

### Procedure

Participants were tested in person in a laboratory room at University College London. All participants gave informed written consent. Demographic data were then collected, which included questions on their highest level of education achieved, age of learning Chinese and English, other known languages and languages used at school or work and at home. Participants began by completing the Chinese LexTALE followed by the English LexTALE.

Participants were then given instructions regarding the eye-tracking section of the study (see Online Appendix E https://osf.io/2va7x/files/) and were set up on the eye-tracker by completing a 3-point calibration and validation procedure, which was repeated until the average error was below 0.30. Participants completed 9 practice trials before the experimental trials to familiarise themselves with the task (see Online Appendix D https://osf.io/2va7x/files/). The order of presentation of the experimental trials was randomised for each participant to avoid systematic order effects. Along with the experimental trials, each participant was also presented with comprehension statements following 40 sentences (a third of the trials). Participants responded to these comprehension statements by pressing the “z” key on the keyboard for true statements and the “/” key for false statements. Participants were given a break every 15 trials to reduce effects of fatigue and the calibration and validation procedure was repeated after every break to ensure good quality tracking. The entire study lasted under an hour and participants were fully debriefed after the study.

### Data analysis

#### Data cleaning and final sample

Following our pre-registered exclusion criteria, of the 62 participants recruited to participate in this study, 7 were excluded for incomplete eye-tracking data due to track loss or poor calibration, and 1 was excluded for scoring below chance (< 50%) on the English LexTALE. All participants scored above chance (> 50%) on the LexTale for Chinese proficiency. This resulted in a final sample of 54 Chinese native speakers (50 females and 4 males), with ages ranging from 18 to 30 years old (*M*years = 21.8, *SD*years = 2.40). All participants started learning Chinese before the age of 5; 30 participants began learning English before the age of 5, and 24 started learning English at the average age of 7.6 years.

Again, following the pre-registered criteria, eye movement data were first cleaned using the clean function from Data Viewer. Short fixations (< 80 ms) within a character of a previous or subsequent fixation were merged with the adjacent fixation and, subsequently, fixations less than 80 ms were removed. After importing data into R, we then removed 208 trials that contained more than 5 blinks and a further 46 trials that contained a blink on the target word, leaving a total of 6,226 trials.

For each eye movement measure, outliers for reading times on target words were identified for each subject within each of the three experimental conditions (TL, SL, and ID). According to Hoaglin and Iglewicz (1987), outliers are those that are 2.2 times the difference between the difference above and below the third and first quartiles:

lower boundary = Q1 – 2.2*(Q3–Q1) and upper boundary = Q3 + 2.2*(Q3–Q1), where Q1 refers to the first quartile and Q3 the third quartile.

Based on the above-described procedure and removing trials with single fixations or gaze durations greater than 1,200 ms on the target words as pre-registered,

(1) for gaze duration: 567 trials were inputted as “NA,” resulting in a total of 5,659 trials from the original 6,480 trials,
(2) for go past time: 646 trials were inputted as “NA,” resulting in a total of 5,580 trials from the original 6,480 trials,
(3) for total reading time: 1,353 trials were inputted as “NA,” resulting in a total of 4,873 trials from the original 6,480 trials.

#### Categorising proficiency

**Figure 3.**
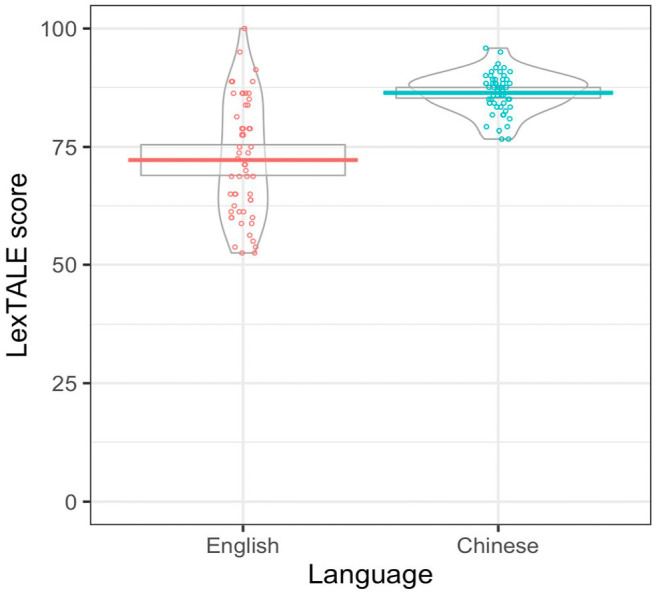
English and Chinese LexTALE scores, where each subject’s score is plotted as points, the horizontal lines display the grand means, the boxes around the lines indicate 95% confidence intervals assuming a normal sampling distribution, and the violins indicate the density.

Figure 3 displays the English LexTALE scores for each participant which, after excluding the participants who scored below 50%, ranged from 52.5% to 100% (*M*_correct_ = 72.2%, *SD*_correct_ = 12.2%). English proficiency bands are reported in Table 2 for participants who learned English before or after the age of 5. There appear to only to be minimal differences in distribution between the groups. As pre-registered, we decided based on the pattern in the scores whether to treat proficiency as a continuous variable, or to treat it as a factor by categorising those scoring in the upper and lower advanced band of the LexTale (80–100%) as high proficiency, and those in the upper intermediate band (60%–79%) as low proficiency. Visual examinations of the overall pattern in the scores as displayed in Figure 3 suggested that it would be more appropriate to collapse the participants into one sample, as we had many more participants scoring in the upper intermediate band. It would also be problematic to suggest that participants scoring 79% and 80% would differ greatly in their proficiency. Hence, we decided to treat proficiency as a continuous between-subject variable. Figure 3 also displays the Chinese LexTALE scores for each participant, which ranged from 76.7% to 95.8% (*M*_correct_ = 86.4%, *SD*_correct_ = 4.2%), suggesting that all participants are highly proficient in Chinese.

**Table 2.** Number of participants scoring in each English LexTALE band for those who learnt English before and after the age of 5.

English LexTALE Band	English before age 5	English after age 5
Lower intermediate (50%–60%)	5	3
Upper intermediate (60%–80%)	8	7
Upper and lower advanced (80%–100%)	17	14

#### Linear mixed-effects analysis

##### Confirmatory analysis

Data were analysed using linear mixed-effects models (LMMs) constructed with the *lme4* package (version 1.1.29; Bates et al., 2015) in *R* (version 4.1.1; R Development Core Team, 2021). We pre-registered an initial model that included a categorical fixed effect of condition where the cont.helmert() function was used to implement the following contrasts:

(1) TL and SL vs. ID, where TL = –1, SL = –1, ID = 2,
(2) TL vs. SL, where TL = –1, SL = 1, ID = 0.

The first contrast compares reading time in our nonword condition (TL + SL)/2 to reading times in our identity condition. This provides an index of the cost associated with processing nonwords. The second contrast compares reading times on TL and SL nonwords, and provides an estimate of whether there is less of a cost for TL nonwords.

The model included a fixed-effect of condition, with three levels, and participants and items were included as random effects: *lmer(log(dv)~ condition + (1 | participant)+ (1 | item))*. The eye movement measures were log-transformed to reduce the rightwards skew of the data. The structure of the participant and item random effects was determined for the model using the buildmer() function from the buildmer package (Voeten, 2019). This function automates the fitting procedure of the random effects structure by identifying the maximal model that converges and performing backward stepwise elimination based on changes in model fit such as log-likelihood, Akaike information criterion (AIC), Bayesian information criterion (BIC), and changes in explained deviance. Model selection criteria may be at risk of over-fitting when based on the AIC but may be at risk of under-fitting when based on the BIC. Hence, when taken together, they reflect how well model parameters/complexity fit the data.

#### Exploratory analysis

In addition to our pre-registered model, we fitted a supplemental model to compare reading time measures between ID and TL target words. Within this model, *lmer(log(dv)~ condition + (1 | participant)+ (1 | item))*, ID was coded as 1 and TL was coded –1. The approach to determining random slopes was identical to our pre-registered analysis.

To evaluate the role of proficiency, we pre-registered an additional model that included an interaction of each of the initial model contrasts with proficiency (i.e., the English LexTALE scores). The same approach for the initial model was used, where a categorical fixed effect of condition included three levels with the same contrasts and coding, and participants and items were included as random effects as determined using buildmer(): *lmer(log(dv)~ condition × proficiency + (1 | participant)+ (1 | item))*. The model thus includes predictors of condition and proficiency, as well as the interaction between these two variables. The English LexTALE scores were scaled and centred prior to fitting these models.

#### Bayes factors

We also supplemented each of our pre-registered frequentist analyses with Bayes Factor analyses, which evaluate the evidence for critical null effects (Wagenmakers et al., 2017). We computed Bayes Factors by first fitting Bayesian LMMs with the same structure as the *lmer* models, using the *brm()* function from the *brms* package (version 2.18.0; Bürkner, 2017). Priors for the fixed effects of condition were set to model estimates using data from Cong and Chen (2022) and non-informative priors normal(0,1) were assumed for other fixed effects. Each model used 12,000 iterations with four chains, of which the first 2,000 iterations were discarded as warm-up. The *hypothesis()* function was then used to calculate the Bayes Factors (BF10) for each fixed effect. BFs greater than 3 are considered evidence against the null (with BF > 10 constituting strong evidence), while BFs < 1/3 are considered evidence for the null. BFs between 1/3 and 3 constitute ambiguous evidence (Jeffreys, 1961).

## Results

### Probability of fixation

On average, probability of fixation for each type of target word was 0.95 (Table 3). Hence, skipping rate was low, which may be due to the relatively long length of the target words.

**Table 3.** Probability of fixation for each target word condition.

Word condition	*Probability of fixation (SD)*
Transposed (TL)	0.95 (0.23)
Substituted (SL)	0.96 (0.20)
Identity (ID)	0.94 (0.23)

### Comprehension accuracy

Mean comprehension accuracy was 93.8% (range = 77.5%–100%, *SD* = 5.33%). All scores were above the 65% lower limit for exclusion, the same threshold used by Cong and Chen (2022), indicating that participants carefully read and understood the sentences. Hence, no further participants were excluded based on this criterion.

### Letter-position coding across the whole sample

#### Confirmatory analysis

**Figure 4.**
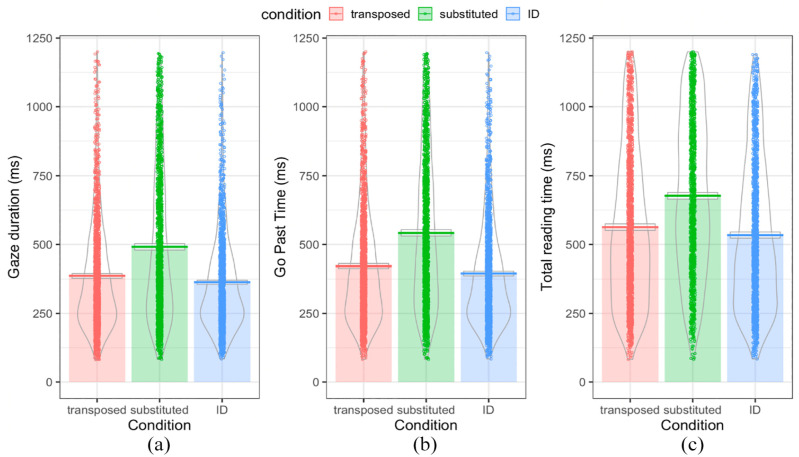
(a) Gaze duration, (b) Go past times, and (c) Total reading times for target words in each of the three conditions: transposed, substituted, and ID, where the raw data is plotted as points, the bars and horizontal line display the means, the boxes around the line indicate 95% confidence intervals assuming a normal sampling distribution, and the violins indicate the density.

Data are visualised in Figure 4. Table 4 displays LMM summary statistics and Bayes Factors for the partial replication of Cong and Chen (2022), with contrasts of condition (TL and SL vs. ID, and TL vs. SL), as specified. For each of the outcome eye movement measures, our pre-registered model fitted to log-transformed data converged with intercept-only structures (as determined by *buildmer*) for the random effects was: *lmer(log(dv)~ condition + (1 | participant)+ (1 | item))*.

**Table 4.** Linear mixed-effect model outcomes for all dependent measures: gaze duration, go past time, and total reading time, with predictors of manipulation (TL and SL vs ID) and manipulation type (TL vs SL).

	Gaze Duration					Go Past Time					Total Reading Time				
Predictors	Estimates	*SE*	t	p	BF10	Estimates	*SE*	t	p	BF10	Estimates	*SE*	t	p	BF10
(Intercept)	5.88	0.03	203.98	**< 0.001**		5.98	0.03	173.03	**< 0.001**		6.31	0.03	188.35	< **0.001**	
Condition (TL + SL vs ID)	–0.05	0.00	–12.57	**<0.001**	Inf	–0.07	0.00	–16.80	**<0.001**	Inf	–0.07	0.00	–14.56	<**0.001**	Inf
Condition (TL vs SL)	0.11	0.01	14.70	**<0.001**	Inf	0.13	0.01	18.63	**<0.001**	Inf	0.13	0.01	15.00	<**0.001**	Inf

*Note*. Significant effects are indicated in bold. *SE*: standard error, BF10: Bayes Factors; TL: transposed letter; SL: substituted letter; ID: identity.

For all eye movement measures, the first contrast within the pre-registered model indicated that reading times were significantly shorter on ID targets relative to the mean of TL and SL targets. The second contrast indicated that reading times were significantly longer on SL targets relative to TL targets. Together, this indicates that both initial and later encoding were shortest for ID targets and that SL targets took longer to encode than TL targets. Based on available evidence for each of these measures, Bayes Factors (BF10) also indicated strong evidence against the null for the effects of condition on all reading time measures. These results indicate that, consistent with the pre-registered hypotheses, Chinese native speakers demonstrate flexible letter-position encoding in the initial and later processing stages, spending significantly more time looking at the SL nonwords than the TL nonwords.

### Exploratory analysis

Visual inspection of Figure 4 suggested minimal differences between TL and ID target words, especially for gaze duration, which raised the question of whether participants processed letter-position at all for the internal letters. Hence, extra contrasts comparing TL and ID target word reading times were run. Summary statistics and Bayes Factors of these models are displayed in Table 5. For each of the outcome eye movement measures, our model fitted to log-transformed data converged with intercept-only structures (as determined by *buildmer*) for the random effects was: *lmer(log(dv)~ condition + (1 | participant)+ (1 | item))*.

**Table 5.** Linear mixed-effect model outcomes for all dependent measures: gaze duration, go past time, total reading time, with predictor TL vs ID.

	Gaze Duration					Go Past Time					Total Reading Time				
Predictors	Estimates	*SE*	*t*	*p*	BF10	Estimates	*SE*	*t*	*p*	BF10	Estimates	*SE*	*t*	*p*	BF10
(Intercept)	5.80	0.03	203.70	**<0.001**		5.88	0.03	175.11	**<0.001**		6.22	0.04	176.95	<**0.001**	
Condition (TL vs ID)	–0.03	0.01	–3.82	**<0.001**	9,999	–0.04	0.01	–5.44	**<0.001**	Inf	–0.04	0.01	–4.74	<**0.001**	Inf

*Note*. Significant effects are indicated in bold. *SE*: standard error, BF10: Bayes Factors; TL: transposed letter; ID: identity.

For all eye movement measures, LMM models indicated that there was a significant difference between TL vs. ID conditions. Based on available evidence for each of these measures, Bayes Factors (BF10) also indicated strong evidence against the null for this effect of condition on reading times. These results emphasise that participants did process letter-position for medial letters in initial and later stages of processing, as they spent significantly more time looking at the TL word over the ID word.

### Proficiency and flexible letter-position coding

#### Exploratory analysis

**Figure 5.**
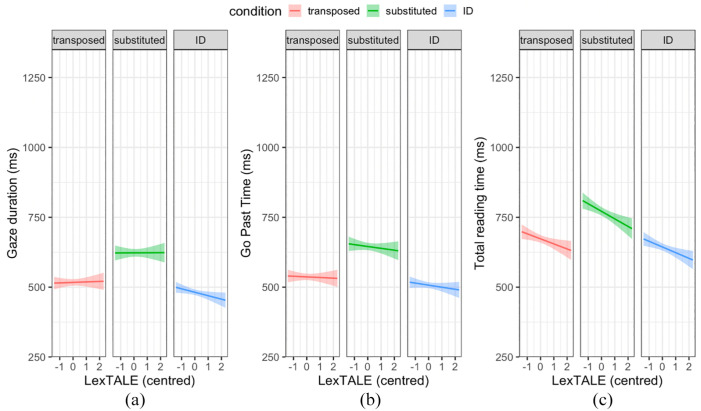
(a) Gaze duration, (b) Go past times, and (c) Total reading times for target words in each condition (transposed, substituted and ID) plotted against the participants’ corresponding scaled and centred English LexTALE scores. Solid lines indicate the linear relationship, and shaded areas indicate 95% confidence intervals.

Our planned exploratory analyses evaluated the interaction between each of the pre-registered contrasts of the three conditions (TL, SL, ID) and proficiency (English LexTALE) on reading time measures. Figure 5 displays the relationship between each of the three reading time measures and English LexTALE scores for the three conditions. Visual inspection of these figures suggested an overall negative trend between reading times and LexTALE scores but minimal differences in slopes between the conditions.

Table 6 displays summary statistics and Bayes Factors of the LMM models with contrasts of condition (TL and SL vs. ID, and TL vs. SL) and proficiency, as well as the interactions between contrasts of condition and proficiency, as specified. For each of the outcome eye movement measures, our model fitted to log-transformed data converged with intercept-only structures (as determined by *buildmer*) for the random effects was: *lmer(log(dv)~ condition × proficiency + (1 | participant)+ (1 | item))*.

**Table 6.** Linear mixed-effect model outcomes for all dependent measures: gaze duration, go past time, total reading time, with predictors of manipulation (TL and SL vs ID), manipulation type (TL vs SL), English LexTALE, interaction between manipulation and English LexTALE, and the interaction between manipulation type and English LexTALE.

	Gaze Duration					Go Past Time						Total Reading Time			
Predictors	Estimates	*SE*	*t*	*p*	BF10	Estimates	*SE*	*t*	*p*	BF10	Estimates	*SE*	*t*	*p*	BF10
(Intercept)	5.89	0.03	203.13	**<0.001**		5.99	0.03	173.13	**<0.001**		6.32	0.03	189.85	**<0.001**	
Condition (TL + SL vs ID)	–0.05	0.00	–12.18	**<0.001**	Inf	–0.07	0.00	–16.40	**<0.001**	Inf	–0.07	0.00	–14.17	**<0.001**	Inf
Condition (TL vs SL)	0.11	0.01	14.39	<**0.001**	Inf	0.13	0.01	18.24	**<0.001**	Inf	0.13	0.01	14.68	**<0.001**	Inf
English LexTALE	–0.03	0.03	–1.16	0.246	6.704	–0.05	0.03	–1.36	0.174	10.399	–0.05	0.03	–1.69	0.092	19.314
Condition (TL + SL vs ID)*English LexTALE	–0.01	0.00	–1.90	0.057	0.028	–0.01	0.00	–1.52	0.129	0.014	–0.00	0.00	–0.91	0.362	0.007
Condition (TL vs SL)*English LexTALE	0.01	0.01	1.01	0.312	0.013	0.01	0.01	1.04	0.299	0.013	0.00	0.01	0.49	0.627	0.010

*Note*. Significant effects are indicated in bold. *SE*: standard error, BF10: Bayes Factors; TL: transposed letter; SL: substituted letter; ID: identity.

For all eye movement measures, the pre-registered models fitted to log-transformed data indicated that simple effects of condition on each eye movement measure were significant, as in the original pre-registered models that did not include proficiency, and Bayes Factors (BF10) again supported this. Despite the seemingly negative relationship between reading times and LexTALE scores seen in the figures, the simple effect of English LexTALE was non-significant in the pre-registered LMM models. However, the Bayes Factor analyses suggested that there was evidence against the null for this simple effect for all reading time measures (all BF10s > 3). Regarding the predicted interactions between TL + SL vs. ID and English LexTALE, and between TL vs. SL and English LexTALE, these were non-significant. Bayes Factor analyses also indicated that there was evidence for the null for these interactions for all reading time measures (all BF10s < 1/3). Overall, these models suggest that more proficient readers had faster reading times, but that the frequentist analyses lacked the power to detect this effect. However, they suggest that proficiency did not significantly influence the difference in reading times between TL, SL, and ID target words.

## Discussion

This pre-registered study investigated whether native Chinese speakers adopt flexible letter-position coding in their L2 English sentence reading and explored whether this depends on their level of English proficiency. To address these research questions, we partially replicated Cong and Chen’s (2022) single-sentence eye-tracking study using their within-morpheme TL and SL conditions. This meant that we could focus on letter-position and identity effects in a well-powered study. Participants completed the English LexTALE as a measure of English proficiency. Consistent with pre-registered hypotheses 1 and 2, our findings indicated that Chinese native speakers demonstrate flexible letter-position coding in the initial and later processing stages of word reading (longer looking times for SL than TL targets, and for these nonword relative to ID targets). Additional analyses also confirmed that participants did process letter-position for medial letters in both initial and later stages of processing (longer looking times for TL than ID targets). Regarding pre-registered hypotheses 3 and 4, our exploratory analyses obtained a non-significant main effect of proficiency and no interaction between proficiency and the condition contrasts, for all reading time measures. For the interaction of proficiency with condition, the Bayes Factor analyses supported this conclusion. Thus, proficiency did not influence the difference in reading times between different target word types, suggesting that proficient L2 participants did not adopt different letter-position coding depending on their level of English proficiency. However, for the main effect of proficiency, Bayes Factors suggested that there was evidence against the null. It therefore seems likely that higher English proficiency was associated with shorter overall reading times, but that we lacked the power to detect this effect in the frequentist statistics. Our novel contributions can therefore be summarised as follows: (1) replication of the within-morpheme condition from Cong and Chen (2022), supporting the idea that Chinese native speakers adopt flexible letter-position coding when reading in L2 English, (2) no evidence that proficiency influences letter-position coding in proficient L2 readers, (3) weak evidence that proficiency, as measured by the LexTALE, influences overall word reading time. We discuss these contributions in the following sections.

### Replication of Cong and Chen (2022)

Our findings indicated that Chinese native speakers demonstrate flexible letter-position coding in their L2 English sentence reading. Reading times indicative of both initial and later processing stages were significantly longer for SL than TL target nonwords. This replicates the within-morpheme condition from Cong and Chen (2022). It suggests that L2 English speakers may process written words in the same way as native English speakers, as findings are consistent with priming studies in native English speakers that found TL primes to significantly facilitate the processing of target words compared to SL primes (Ziegler et al., 2014). Reading time measures in our experiment were also significantly longer for the average of SL and TL target nonwords compared to ID real words, emphasising that manipulation of the ID words interferes with reading as hypothesised and again replicating Cong and Chen (2022).

Additional contrasts showed that all reading time measures were significantly longer for TL than ID target words, highlighting that despite some flexibility, participants do code letter-position during L2 single-sentence reading. This contrasts with Cong and Chen (2022), who reported no difference in reading times between TL and ID targets, which would suggest that individuals do not process medial letter-positions but rather only letter identity. Though the TL vs. ID contrast was not pre-registered, our study provided increased power and sample size relative to Cong and Chen. Power simulations suggested that 21 participants would be sufficient to replicate within-morpheme findings from Cong and Chen (2022) with at least ~90% power for both pre-registered contrasts of TL and SL vs. ID and TL vs. SL. Far exceeding this, our final sample included 54 participants due to over-recruitment for exploratory analyses. Our exploratory findings therefore suggest that, though they encode letter-position flexibly, Chinese-English bilinguals do process medial letter-positions during English sentence reading. This again mirrors findings in priming studies with native English speakers (Kezilas et al., 2017), as well as eye-tracking studies showing that TL nonwords have longer reading times than target words in natural sentence reading. Findings are also consistent with studies with Chinese-English bilinguals that used single-word presentation and mouse-tracking (Lin & Lin, 2016) as well as masked priming (Chen et al., 2020; Lei et al., 2021).

### Null effects of proficiency on letter-position coding

We predicted bigger differences in reading times between the conditions (particularly SL > TL) for more proficient English speakers. Findings were not consistent with this hypothesis, as native Chinese speakers’ English proficiency did not interact with condition for any of the reading time measures, with evidence for the null from Bayes Factor analyses. Thus, letter-position coding was not more flexible for participants with higher English proficiency.

A few previous studies using priming and single-word presentation techniques have observed differences in the TL effect depending on proficiency or experience. Chen et al. (2020) used a forward-masked English lexical decision task to look at TL effects in two groups of adult Chinese-speaking English learners, of whom 30 were high and 30 low English proficiency. TL primes significantly facilitated target word processing compared to SL primes. The size of this effect did not differ according to proficiency but was only significant for high, and not low, frequency targets. This suggests that flexible letter-position coding depends on the amount of exposure to individual words, rather than overall proficiency levels. Lei et al. (2021) also used a forward-masked English lexical decision task with Chinese native speakers, of whom 10 were low and 10 intermediate English proficiency. They observed that higher frequency words and higher proficiency readers showed a greater TL effect. Andrews and Lo (2012) examined individual differences in forward-masked priming effects on lexical decision in a sample of 100 undergraduate participants. There was stronger inhibition from TL nonword primes and stronger facilitation from close neighbour pseudoword primes, for participants with higher scores on an individual differences measure that captured the shared variance among reading, spelling, and vocabulary.

Overall, several studies with adult readers have observed that proficiency and/or experience influences the specificity with which written words are encoded in the lexicon. Thus, our failure to find such effects is inconsistent with both studies on individual differences and with some studies examining maturational differences (e.g., Grainger et al., 2012; Ziegler et al., 2014). These discrepancies may be due to differences in experimental tasks. Sentence reading paradigms, like the one we used, provide meaningful context and examine processing over multiple fixations, unlike isolated word presentation paradigms such as lexical decision (Blythe et al., 2014). Future research could examine the sensitivity of both paradigms in eliciting proficiency effects. It could also examine earlier stages of processing in parafoveal areas. Findings from Cong and Chen (2022) suggest that L2 readers struggle to extract and utilise identity and position information of parafoveal letters in a word. Replication of such findings would enable a wider understanding of how Chinese-English bilinguals process words in L2 sentence reading. The fact that we observed a significant TL vs. ID contrast, whereas Cong and Chen did not, may also be due to our sample being more proficient, which is evident in the shorter total reading times seen in our study (~500–700 ms) as compared to Cong and Chen (> 1,000 ms). This is likely because our study was conducted at a London university, whereas Cong and Chen’s (2022) experiment was conducted in China and, hence, our sample likely had more extensive experience in reading English.

Another issue is that sample sizes in previous studies of proficiency and letter-position coding have been very varied and observed estimates have often been very small. Well-powered replications that can detect small effects are therefore necessary before we can be confident about previously reported proficiency effects (Andrews & Lo, 2012; Kahraman & Kırkıcı, 2021; Veldre & Andrews, 2014). Our study may have also suffered from a lack of power to detect interactions between proficiency and letter-position coding and, though pre-registered, these analyses were therefore deemed exploratory. Furthermore, we conducted reliability analyses (see Supplementary Materials), which indicated that low power was compounded by a lack of within-subject reliability in the difference in reading times for TL versus SL targets, though the overall difference between TL and SL versus ID targets was more reliable. It is difficult to optimise a study design to reliably measure both the effect of an experimental variable within-participant (e.g., TL vs. SL reading times) and the relationship between such a measure and a between-participant variable (such as reading proficiency; Blott et al., 2023; Goodhew & Edwards, 2019). This is because design choices often work against each other, such as whether to counterbalance experimental lists and whether to prioritise between-participant variation vs. between-condition differences. It is also often difficult to separate overall task performance from the individual differences in a specific process that are of most interest. Future work investigating the development of letter-position coding in developing and non-native language readers will need to consider these issues if progress is to be made.

### Weak effects of proficiency on overall reading times

We predicted that overall target word reading times would be negatively related to proficiency. In our pre-registered frequentist analyses this prediction was not confirmed. However, Figure 5 suggests that the predicted relationship was present, and this was supported by Bayes Factor analyses which indicated evidence against the null for this effect for all reading time measures (BF > 3), with this evidence being strong for go past and total reading times (BF > 10). This suggests that proficiency as measured by the LexTALE does index something that reliably relates to reading times as measured with eye-tracking during sentence reading, which is reassuring. This is consistent with previous studies using priming and single-word presentation techniques in non-native English speakers. For example, both Chen et al. (2020) and Lei et al. (2021) observed longer overall response times in a forward-masked English lexical decision task for low relative to high English proficiency Chinese native speakers. The non-significant effect in our frequentist analysis was therefore likely due to a lack of power.

Future studies should consider how best to measure English proficiency. We used the English LexTALE, which indexes the ability to discriminate between words and nonwords. This task is a good predictor of English vocabulary knowledge and correlates highly with general English proficiency measures like the Quick Placement Test, and more so than self-ratings of proficiency (Lemhöfer & Broersma, 2012). Lemhöfer and Broersma (2012) reported split-half reliability ranging from .81 for Dutch participants to .68 for Korean participants. However, our sample mainly scored in the upper intermediate band and the distribution of scores for those who learnt English before and after the age of 5 were indistinguishable, therefore the LexTALE may not have been optimally sensitive to variation within our highly proficient sample. In contrast, Chen et al. (2020) categorised proficiency according to whether participants were an English major at university and Lei et al. (2021) used the Quick Placement Test to determine English proficiency levels within the Common European Framework. Such measures may be more sensitive to current English usage. Furthermore, the English LexTALE only assesses whether individuals recognise written words. A latent variable encompassing reading, spelling, and vocabulary, as used by Andrews and Lo (2012), is arguably a more comprehensive metric. Relatedly, Parker and Slattery (2021) reported that reading but not spelling ability influenced intra-line fixation durations (see also Slattery & Yates, 2018, for a similar pattern in sentence reading). So, the picture of individual differences in eye movement fixation measures is likely more complex than is assessable with a single measure. Future research should examine the influence of different measures of proficiency on letter-position coding, in both foveal and parafoveal vision, to fully understand how proficiency modulates letter-position coding.

### Theoretical implications

Our finding of flexible letter-position coding in Chinese-English L2 bilinguals’ English sentence reading is compatible with current models of visual word recognition including those that incorporate noisy position coding (Davis, 2010; Gomez et al., 2008; Norris, 2006), the LTRS model, which assumes that letter identity and position information is accumulated accurately over time (Adelman, 2011), and the multiple route model (Grainger et al., 2012), which captures the overlap between words with similar letters in different positions using open-bigram coding. In addition, our findings indicate that these models apply regardless of first language or proficiency, though such conclusions must be taken with caution due to issues already discussed. The multiple route model provides an account not just of orthographic coding but also of how readers map from orthography to phonology and semantics. In this model, flexible letter-position coding arises from the coarse-grained orthographic representations implemented in the direct orthography-to-semantics route. Our findings thus suggest that Chinese-English L2 bilinguals encode letters in parallel in a coarse-grained manner and access meaning directly from print, rather than processing words letter-by-letter and accessing meaning via phonology. Also consistent with the multiple route model, we found that participants spent more time fixating on TL nonwords than ID words, indicating that information about medial letter-position, not just identity, was processed by our Chinese-English bilingual sample.

As reviewed in the Introduction, previous studies investigating the development of flexible letter-position coding have compared different age groups. Several studies suggest that the benefit of TL relative to SL primes increases with age (Grainger et al., 2012; Ziegler et al., 2014), though there are also some inconsistent results (e.g., Acha & Perea, 2008; Hasenäcker & Schroeder, 2022; Kezilas et al., 2017). However, these findings may arise from increased experience with words or more general effects of cognitive or perceptual maturation. Observing proficiency effects on written word representations within an L2 adult sample would provide stronger evidence for the role of experience. Though we did not observe such effects, our findings do suggest that coarse-grained orthographic coding, indicative of efficient access to meaning from print, is evident in relatively proficient second-language learners.

In second-language learners, we must also consider the effect of first-language orthography on the development of their second-language letter-position coding. Studying Chinese-English bilinguals may avoid this issue to a certain extent, since Chinese is not an alphabetic script (Chen et al., 2020; Cong & Chen, 2022). However, the nature of character-position coding in Chinese may still be relevant. Yang et al. (2020) found that, unlike readers of alphabetic orthographies, native Chinese readers do not adopt precise character-position coding as they demonstrated strong backward priming in their L1 (e.g., *DCBA* primes *ABCD*). Yang et al. (2021) further demonstrated that Chinese-English bilinguals exhibited strong backward priming in an L2 English lexical decision task, where backward primes such as “*yalp”* would facilitate the target word “*play.*” Such an effect was not present in English monolinguals, nor was it displayed in Spanish-English or Arabic-English bilinguals. This suggests that position is coded more coarsely in Chinese readers than in readers of alphabetic scripts. Further supporting evidence comes from Pae et al. (2017) who found that scrambled letters interfered less with English word naming in Chinese-English speakers compared to Korean-English and English native speakers. Future research should aim to understand letter-position coding at different levels of proficiency within and between speakers of different native languages.

Future research should also consider whether the multiple route model (Grainger et al., 2012), which was developed to account for monolingual learning of alphabetic scripts, is a suitable model for bilingual reading. Current bilingual word recognition models, such as the bilingual IA (Dijkstra et al., 1998) and bilingual IA plus (Dijkstra & van Heuven, 2002) models, assume position-specific letter-position coding. This is incompatible with the current findings and previous literature (e.g., Chen et al., 2020; Cong & Chen, 2022; Lei et al., 2021). The more recent Multilink model (Dijkstra et al., 2019), which focuses on foveal word recognition, attempts to bypass issues with strict position-specific coding by skipping sub-lexical levels and using the Levenshtein distance as a measure of orthographic similarity between lexical items. However, the lack of letter representations in this model means that it cannot provide a full account of how flexible letter-position coding in L2 learners, as demonstrated in the current study, is achieved. Thus, further research is needed in developing an appropriate model of L2 reading that captures the potential transition from serial to parallel orthographic representations, as well as the influence of L1 on L2 letter-position coding.

## Conclusion

In summary, using a subset of the experimental stimuli from Cong and Chen (2022), we replicated their findings, indicating that native Chinese speakers adopt flexible letter-position coding during L2 English sentence reading, spending more time fixating SL than TL target nonwords. In addition, and in contrast to Cong and Chen (2022), we also found that native Chinese speakers process medial letter-position as they spent more time fixating TL nonword than ID word targets. Our well-powered and pre-registered partial replication, therefore, provides further evidence regarding the presence of flexible letter-position coding, indicative of direct print-to-meaning access, in L2 speakers of English. Considering proficiency effects among our highly proficient sample, though our pre-registered frequentist analyses did not find significant effects of English LexTALE score on overall reading times, Bayes Factors provided evidence against the null in the predicted direction. This suggests that the LexTALE and word reading times, as measured by eye-tracking during sentence reading, may be indexing a common component of reading/language ability. In contrast, we did not find that proficiency influenced letter-position coding. However, these analyses were compromised by low statistical power and reliability of the experimental contrast of most interest. This is also an issue with other research in this field, therefore, further well-powered research is necessary. This work will need to consider which measures of proficiency and which experimental designs are most appropriate for reliably examining individual differences in letter-position processing.

## Supplemental Material

sj-docx-1-qjp-10.1177_17470218241229442 – Supplemental material for Flexible letter-position coding in Chinese-English L2 bilinguals: Evidence from eye movements Supplemental material, sj-docx-1-qjp-10.1177_17470218241229442 for Flexible letter-position coding in Chinese-English L2 bilinguals: Evidence from eye movements by Hillarie Man, Adam J Parker and J. S. H. Taylor in Quarterly Journal of Experimental Psychology

## References

[bibr1-17470218241229442] Acha J. Perea M. (2008). The effects of length and transposed-letter similarity in lexical decision: Evidence with beginning, intermediate, and adult readers. British Journal of Psychology, 99(2), 245–264. 10.1348/000712607X224478 17631694

[bibr2-17470218241229442] Adelman J. S. (2011). Letters in time and retinotopic space. Psychological Review, 118(4), 570–582. 10.1037/A0024811 21823804

[bibr3-17470218241229442] Andrews S. Lo S. (2012). Not all skilled readers have cracked the code: Individual differences in masked form priming. Journal of Experimental Psychology: Learning Memory and Cognition, 38(1), 152–163. 10.1037/A0024953 21875252

[bibr4-17470218241229442] Anwyl-Irvine A. L. Massonnié J. Flitton A. Kirkham N. Evershed J. K. (2020). Gorilla in our midst: An online behavioral experiment builder. Behavior Research Methods, 52(1), 388–407. 10.3758/S13428-019-01237-X/TABLES/8 31016684 PMC7005094

[bibr5-17470218241229442] Bates D. Mächler M. Bolker B. M. Walker S. C. (2015). Fitting linear mixed-effects models using lme4. Journal of Statistical Software, 67(1), 1–48. 10.18637/jss.v067.i01

[bibr6-17470218241229442] Blott L. M. Gowenlock A. E. Kievit R. Nation K. Rodd J. M. (2023). Studying individual differences in language comprehension: The challenges of item-level variability and well-matched control conditions. Journal of Cognition, 6(1), Article 54. 10.5334/JOC.317 PMC10487189 37692192

[bibr7-17470218241229442] Blythe H. I. Johnson R. L. Liversedge S. P. Rayner K. (2014). Reading transposed text: Effects of transposed letter distance and consonant-vowel status on eye movements. Attention, Perception, and Psychophysics, 76(8), 2424–2440. 10.3758/S13414-014-0707-2 24980151

[bibr8-17470218241229442] Bürkner P. C. (2017). Brms: An R package for Bayesian multilevel models using Stan. Journal of Statistical Software, 80, 1–28. https://doi/org/10.18637/jss.v080.i01

[bibr9-17470218241229442] Castles A. Rastle K. Nation K. (2018). Ending the reading wars: Reading acquisition from novice to expert. Psychological Science in the Public Interest, 19(1), 5–51. 10.1177/1529100618786959 29890888

[bibr10-17470218241229442] Chan I. L. Chang C. B. (2018). LEXTALE_CH: A quick, character-based proficiency test for Mandarin Chinese. In Bertolini A.B. Kaplan M.J. (Eds.) Proceedings of the 42nd Annual Boston University Conference on Language Development Cascadilla Press (pp. 114–130).

[bibr11-17470218241229442] Chen Y. Liu H. Yu M. Dang J. (2020). The development on transposed-letter effect in English word recognition: Evidence from Late unbalanced Chinese-English bilinguals. Lingua, 235, Article 102777. 10.1016/J.LINGUA.2019.102777

[bibr12-17470218241229442] Choi S. Gopnik A. (1995). Early acquisition of verbs in Korean: A cross-linguistic study. Journal of Child Language, 22(3), 497–529. 10.1017/S0305000900009934 8789512

[bibr13-17470218241229442] Cong F. Chen B. (2022). The letter position coding mechanism of second language words during sentence reading: Evidence from eye movements. Quarterly Journal of Experimental Psychology, 75(10), 1932–1947. 10.1177/17470218211064539 34806482

[bibr14-17470218241229442] Davis C. J. (2010). The spatial coding model of visual word identification. Psychological Review, 117(3), 713–758. 10.1037/A0019738 20658851

[bibr15-17470218241229442] DeBruine L. M. Barr D. J. (2021). Understanding mixed-effects models through data simulation. Advances in Methods and Practices in Psychological Science, 4(1), 1–15. 10.1177/2515245920965119

[bibr16-17470218241229442] Dijkstra T. van Heuven W. J. B. (2002). The architecture of the bilingual word recognition system: From identification to decision. Bilingualism: Language and Cognition, 5(3), 175–197. 10.1017/S1366728902003012

[bibr17-17470218241229442] Dijkstra T. van Heuven W. J. B. Grainger J. (1998). Simulating cross-language competition with the bilingual interactive model. Psychologica Belgica, 38(3–4), 177–196.

[bibr18-17470218241229442] Dijkstra T. O. N. Wahl A. Buytenhuijs F. Van Halem N. Al-Jibouri Z. De Korte M. Rekké S. (2019). Multilink: A computational model for bilingual word recognition and word translation. Bilingualism, 22(4), 657–679. 10.1017/S1366728918000287

[bibr19-17470218241229442] Gentner D. (1982). Why nouns are learned before verbs: Linguistic relativity versus natural partitioning. Language, 2, 301–334.

[bibr20-17470218241229442] Godfroid A. Ahn J. Choi I. Ballard L. Cui Y. Johnston S. Lee S. Sarkar A. Yoon H. J. (2018). Incidental vocabulary learning in a natural reading context: An eye-tracking study. Bilingualism: Language and Cognition, 21(3), 563–584. 10.1017/S1366728917000219

[bibr21-17470218241229442] Gomez P. Marcet A. Perea M. (2021). Are better young readers more likely to confuse their mother with their mohter? Quarterly Journal of Experimental Psychology, 74(9), 1542–1552. 10.1177/17470218211012960/ASSET/IMAGES/10.1177_17470218211012960-IMG3.PNG 33845705

[bibr22-17470218241229442] Goodhew S. C. Edwards M. (2019). Translating experimental paradigms into individual-differences research: Contributions, challenges, and practical recommendations. Consciousness and Cognition, 69, 14-25. 10.1016/j.concog.2019.01.008 30685513

[bibr23-17470218241229442] Gomez P. Ratcliff R. Perea M. (2008). The overlap model: A model of letter position coding. Psychological Review, 115(3), 577–600. 10.1037/A0012667 18729592 PMC2597794

[bibr24-17470218241229442] Grainger J. (2018). Orthographic processing: A “mid-level” vision of reading: The 44th Sir Frederic Bartlett Lecture. Quarterly Journal of Experimental Psychology, 71(2), 335–359. 10.1080/17470218.2017.1314515 28376655

[bibr25-17470218241229442] Grainger J. Granier J. P. Farioli F. Van Assche E. Van Heuven W. J. B. (2006). Letter position information and printed word perception: The relative-position priming constraint. Journal of Experimental Psychology: Human Perception and Performance, 32(4), 865–884. 10.1037/0096-1523.32.4.865 16846285

[bibr26-17470218241229442] Grainger J. Lété B. Bertand D. Dufau S. Ziegler J. C. (2012). Evidence for multiple routes in learning to read. Cognition, 123(2), 280–292. 10.1016/J.COGNITION.2012.01.003 22357323

[bibr27-17470218241229442] Hasenäcker J. Schroeder S. (2022). Transposed and substituted letter effects across reading development: A longitudinal study. Journal of Experimental Psychology: Learning Memory and Cognition, 48(8), 1202–1218. 10.1037/XLM0001064 34498901

[bibr28-17470218241229442] Hoaglin D. C. Iglewicz B. (1987). Fine-tuning some resistant rules for outlier labeling. Journal of the American Statistical Association, 82(400), 1147–1149. 10.1080/01621459.1987.10478551

[bibr29-17470218241229442] Jeffreys H. (1961). Theory of probability (3rd ed.). Oxford University Press.

[bibr30-17470218241229442] Johnson R. L. Perea M. Rayner K. (2007). Transposed-letter effects in reading: Evidence from eye movements and parafoveal preview. Journal of Experimental Psychology: Human Perception and Performance, 33(1), 209–229. 10.1037/0096-1523.33.1.209 17311489

[bibr31-17470218241229442] Kahraman H. Kırkıcı B. (2021). Letter transpositions and morphemic boundaries in the second language processing of derived words: An exploratory study of individual differences. Applied Psycholinguistics, 42(2), 417–446. 10.1017/S0142716420000673

[bibr32-17470218241229442] Kezilas Y. McKague M. Kohnen S. Badcock N. A. Castles A. (2017). Disentangling the developmental trajectories of letter position and letter identity coding using masked priming. Journal of Experimental Psychology: Learning Memory and Cognition, 43(2), 250–258. 10.1037/XLM0000293 27428877

[bibr33-17470218241229442] Ktori M. Kingma B. Hannagan T. Holcomb P. J. Grainger J. (2014). On the time-course of adjacent and non-adjacent transposed-letter priming. Journal of Cognitive Psychology, 26(5), 491–505. 10.1080/20445911.2014.922092 25364497 PMC4214856

[bibr34-17470218241229442] Lee J. N. Naigles L. R. (2005). The input to verb learning in mandarin Chinese: A role for syntactic bootstrapping. Developmental Psychology, 41(3), 529–540. 10.1037/0012-1649.41.3.529 15910160

[bibr35-17470218241229442] Lei H. Dang J. Chen Y. (2021). An eye-tracking study of transposed-letter effect in English word recognition by Mandarin speakers [Paper presentation]. 12th International Symposium on Chinese Spoken Language Processing (ISCSLP 2021). 10.1109/ISCSLP49672.2021.9362079

[bibr36-17470218241229442] Lemhöfer K. Broersma M. (2012). Introducing LexTALE: A quick and valid Lexical Test for Advanced Learners of English. Behavior Research Methods, 44(2), 325–343. 10.3758/S13428-011-0146-0 21898159 PMC3356522

[bibr37-17470218241229442] Lété B. Fayol M. (2013). Substituted-letter and transposed-letter effects in a masked priming paradigm with French developing readers and dyslexics. Journal of Experimental Child Psychology, 114(1), 47–62. 10.1016/J.JECP.2012.09.001 23046691

[bibr38-17470218241229442] Lin Y. C. Lin P. Y. (2016). Mouse tracking traces the “Camrbidge Unievrsity” effects in monolingual and bilingual minds. Acta Psychologica, 167, 52–62. 10.1016/J.ACTPSY.2016.04.001 27107983

[bibr39-17470218241229442] Liversedge S. P. Paterson K. B. Pickering M. J. (1998). Eye movements and measures of reading time. In Underwood G. (Ed.), Eye guidance in reading and scene perception (pp. 55–75). Elsevier Science. 10.1016/B978-008043361-5/50004-3

[bibr40-17470218241229442] Lupker S. J. Spinelli G. Davis C. J. (2019). Masked form priming as a function of letter position: An evaluation of current orthographic coding models. Journal of Experimental Psychology. Learning, Memory, and Cognition, 46(12), 2349–2366. 10.1037/XLM0000799 31829650

[bibr41-17470218241229442] Martin K. I. Juffs A. (2021). Eye-tracking as a window into assembled phonology in native and non-native reading. Journal of Second Language Studies, 4(1), 65–95. 10.1075/JSLS.19026.MAR/CITE/REFWORKS

[bibr42-17470218241229442] McClelland J. L. Rumelhart D. E. (1981). An interactive activation model of context effects in letter perception: I. An account of basic findings. Psychological Review, 88(5), 375–407. 10.1037/0033-295X.88.5.375 7058229

[bibr43-17470218241229442] Mézière D. C. Yu L. Reichle E. D. von der Malsburg T. McArthur G. (2023). Using eye-tracking measures to predict reading comprehension. Reading Research Quarterly, 58(3), 425–449. 10.1002/RRQ.498

[bibr44-17470218241229442] Norris D. (2006). The Bayesian reader: Explaining word recognition as an optimal Bayesian decision process. Psychological Review, 113(2), 327–357. 10.1037/0033-295X.113.2.327 16637764

[bibr45-17470218241229442] Pae H. K. Kim S. A. Mano Q. R. Kwon Y. J. (2017). Sublexical and lexical processing of the English orthography among native speakers of Chinese and Korean. Reading and Writing, 30(1), 1–24. 10.1007/S11145-016-9660-X/TABLES/2

[bibr46-17470218241229442] Pagán A. Blythe H. I. Liversedge S. P. (2021). The influence of children’s reading ability on initial letter position encoding during a reading-like task. Journal of Experimental Psychology. Learning, Memory, and Cognition, 47(7), 1186–1203. 10.1037/XLM0000989 33539168

[bibr47-17470218241229442] Parker A. J. Slattery T. J. (2021). Spelling ability influences early letter encoding during reading: Evidence from return-sweep eye movements. Quarterly Journal of Experimental Psychology, 74(1), 135–149. 10.1177/1747021820949150 PMC7745609 32705948

[bibr48-17470218241229442] Perea M. Duñabeitia J. A. Carreiras M. (2008). Transposed-letter priming effects for close versus distant transpositions. Experimental Psychology, 55(6), 384–393. 10.1027/1618-3169.55.6.384 19130764

[bibr49-17470218241229442] Perea M. Lupker S. J. (2003). Does jugde activate COURT? Transposed-letter similarity effects in masked associative priming. Memory and Cognition, 31(6), 829–841. 10.3758/BF03196438 14651292

[bibr50-17470218241229442] Perea M. Lupker S. J. (2004). Can CANISO activate CASINO? Transposed-letter similarity effects with nonadjacent letter positions. Journal of Memory and Language, 51(2), 231–246. 10.1016/J.JML.2004.05.005

[bibr51-17470218241229442] Perea M. Mallouh R. A. García-Orza J. Carreiras M. (2011a). Masked priming effects are modulated by expertise in the script. Quarterly Journal of Experimental Psychology, 64(5), 902–919. 10.1080/17470218.2010.512088 20924985

[bibr52-17470218241229442] Perea M. Mallouh R. A. García-Orza J. Carreiras M. (2011b). Masked priming effects are modulated by expertise in the script. Quarterly Journal of Experimental Psychology, 64(5), 902–919. 10.1080/17470218.2010.512088/ASSET/IMAGES/10.1080_17470218.2010.512088-IMG20.PNG 20924985

[bibr53-17470218241229442] Perea M. Marcet A. Gómez P. (2016). How do Scrabble players encode letter position during reading? Psicothema, 28(1), 7–12. 10.7334/PSICOTHEMA2015.167 26820417

[bibr54-17470218241229442] Perea M. Rosa E. Gómez C. (2005). The frequency effect for pseudowords in the lexical decision task. Perception and Psychophysics, 67(2), 301–314. 10.3758/BF03206493 15971693

[bibr55-17470218241229442] Rayner K. Kaiser J. S. (1975). Reading mutilated text. Journal of Educational Psychology, 67(2), 301–306. 10.1037/H0077015

[bibr56-17470218241229442] R Development Core Team. (2021). R: A language and environment for statistical computing. R Foundation for Statistical Computing.

[bibr57-17470218241229442] Share D. L. (1995). Phonological recoding and self-teaching: Sine qua non of reading acquisition. Cognition, 55(2), 151–218. 10.1016/0010-0277(94)00645-2 7789090

[bibr58-17470218241229442] Slattery T. J. Yates M. (2018). Word skipping: Effects of word length, predictability, spelling and reading skill. Quarterly Journal of Experimental Psychology, 71(1 Special Issue), 250–259. 10.1080/17470218.2017.1310264 PMC6159777 28856970

[bibr59-17470218241229442] Stinchcombe E. J. Lupker S. J. Davis C. J. (2012). Transposed-letter priming effects with masked subset primes: A re-examination of the “relative position priming constraint.” Language and Cognitive Processes, 27(4), 475–499. 10.1080/01690965.2010.550928

[bibr60-17470218241229442] Veldre A. Andrews S. (2014). Lexical quality and eye movements: Individual differences in the perceptual span of skilled adult readers. Quarterly Journal of Experimental Psychology, 67(4), 703–727. 10.1080/17470218.2013.826258 23972214

[bibr61-17470218241229442] Voeten C. C. (2019). buildmer: Stepwise elimination and term reordering for mixed-effects regression [Computer software]. Comprehensive R Archive Net work. https://CRAN.R-project.org/package=buildmer

[bibr62-17470218241229442] Wagenmakers E.-J. Verhagen J. Ly A. Matzke D. Steingroever H. Rouder J. N. Morey R. D. (2017). The need for bayesian hypothesis testing in psychological science. Psychological Science Under Scrutiny, 123–138. 10.1002/9781119095910.CH8

[bibr63-17470218241229442] Wang M. Koda K. Perfetti C. A. (2003). Alphabetic and nonalphabetic L1 effects in English word identification: A comparison of Korean and Chinese English L2 learners. Cognition, 87(2), 129–149. 10.1016/S0010-0277(02)00232-9 12590041

[bibr64-17470218241229442] White S. J. Johnson R. L. Liversedge S. P. Rayner K. (2008). Eye movements when reading transposed text: The importance of word-beginning letters. Journal of Experimental Psychology: Human Perception and Performance, 34(5), 1261–1276. 10.1037/0096-1523.34.5.1261 18823209 PMC2662926

[bibr65-17470218241229442] Whitney C. Bertrand D. Grainger J. (2012). On coding the position of letters in words: A test of two models. Experimental Psychology, 59(2), 109–114. 10.1027/1618-3169/A000132 22044790

[bibr66-17470218241229442] Yang H. Hino Y. Chen J. Yoshihara M. Nakayama M. Xue J. Lupker S. J. (2020). The origins of backward priming effects in logographic scripts for four-character words. Journal of Memory and Language, 113, Article 104107. 10.1016/J.JML.2020.104107

[bibr67-17470218241229442] Yang H. Jared D. Perea M. Lupker S. J. (2021). Is letter position coding when reading in L2 affected by the nature of position coding used when bilinguals read in their L1? Memory and Cognition, 49(4), 771–786. 10.3758/S13421-020-01126-1 33469883

[bibr68-17470218241229442] Zeng T. Han B. Zhai M. Mu Y. (2019). The effect of language proficiency on L2 English learners’ processing of morphologically complex words: Evidence from masked transposed letter priming. Neuroscience Letters, 704, 84–88. 10.1016/J.NEULET.2019.03.042 30943429

[bibr69-17470218241229442] Ziegler J. C. Bertrand D. Lété B. Grainger J. (2014). Orthographic and phonological contributions to reading development: Tracking developmental trajectories using masked priming. Developmental Psychology, 50(4), 1026–1036. 10.1037/A0035187 24294878

