# Supplementary material for: Flexible letter-position coding in Chinese-English L2 bilinguals: Evidence from eye movements
Source: Q J Exp Psychol (Hove). 2024 Feb 28;77(12):2497–515. doi: 10.1177/17470218241229442 (PMC11607845; doi:10.1177/17470218241229442)
Supplement: sj-docx-1-qjp-10.1177_17470218241229442 – Supplemental material for Flexible letter-position coding in Chinese-English L2 bilinguals: Evidence from eye movements [file sj-docx-1-qjp-10.1177_17470218241229442.docx]

**Supplementary Materials**

**Reliability of eye movement contrasts**

To check the sensitivity of each of our eye movement measures for detecting individual differences, we calculated the two condition contrasts (TL and SL vs ID, and TL vs SL) separately for odd and even trials for each participant and correlated these (Table 7). Note that in some cases there may not be many trials per condition for each participant after data cleaning and exclusions (the number of trials per condition for each participant ranged from 1 to 40). Parker et al. (2021) argued that 0.65 reliability represents the lower end of the scale for meaningful interpretation of individual differences. Under this threshold, reliability was relatively high for contrast 1, which compared reading times for TL and SL with ID targets, for gaze duration and go past time, but was lower for total reading time. For contrast 2, which compared TL with SL targets, reliability was low. This suggests that aspects of our design were not optimised for individual differences analyses as the absence of a correlation between two measures is only meaningful is tasks are reliable.

Table 7. Pearson correlations of contrasts between odd and even trials across participants for each eye movement measure.

| Predictors | **Gaze Duration** | | **Go Past Time** | | **Total Reading Time** | |
| --- | --- | --- | --- | --- | --- | --- |
|  | N | *Estimates (CI)* | N | *Estimates (CI)* | N | *Estimates (CI)* |
| Condition (TL + SL vs ID) | 54 | 0.70  (0.53-0.82) | 54 | 0.80  (0.68-0.88) | 52 | 0.57  (0.36-0.73) |
| Condition (TL vs SL) | 54 | 0.17  (-0.10-0.42) | 54 | 0.38  (0.13-0.59) | 52 | 0.20  (-0.08-0.45) |

*Note*. CI: 95% confidence intervals.

**Summary table of mean fixation times**

Table 8. Mean and standard deviations of each reading time measure for each condition (TL, SL, ID).

| **Condition** | **Reading time measures (in ms)** | | | | | |
| --- | --- | --- | --- | --- | --- | --- |
|  | **Gaze Duration** | | **Go Past Time** | | **Total Reading Time** | |
|  | ***M*** | ***sd*** | ***M*** | ***sd*** | ***M*** | ***sd*** |
| TL | 381 | 194 | 415 | 205 | 562 | 272 |
| SL | 489 | 268 | 541 | 268 | 677 | 281 |
| ID | 359 | 175 | 389 | 191 | 534 | 264 |

Note. *M*: mean. *sd*: standard deviation. All units are in ms.

**First fixation duration (post-hoc)**

Data is visualised in *Figure 6*. Table 9 displays LMM summary statistics for the partial replication of Cong and Chen (2022), with contrasts of condition (TL and SL vs ID, and TL vs SL), as specified. For first fixation duration, our model fitted to log-transformed data with intercept-only structures (as determined by *buildmer*) for the random effects was *(lmer(log(eye movement measure)~ condition + (1 | participant) + (1 | item))*.

The first contrast within the pre-registered model indicated that reading times were significantly shorter on ID targets relative to the mean of TL and SL targets. The second contrast indicated that first-fixation durations were significantly longer on SL targets relative to TL targets. Together, this indicates that the earliest stages of encoding were shortest for ID targets and that SL targets took longer to encode than TL targets. These results indicate that Chinese native speakers do demonstrate flexible letter-position encoding in the very initial processing stages, spending significantly more time looking at the SL word than the TL word. These findings differ from the data patterns in Cong and Chen (2021) where none of their contrasts for first fixation duration was significant, which could potentially be attributed to differences in proficiency and language experience in our sample compared to theirs.

**
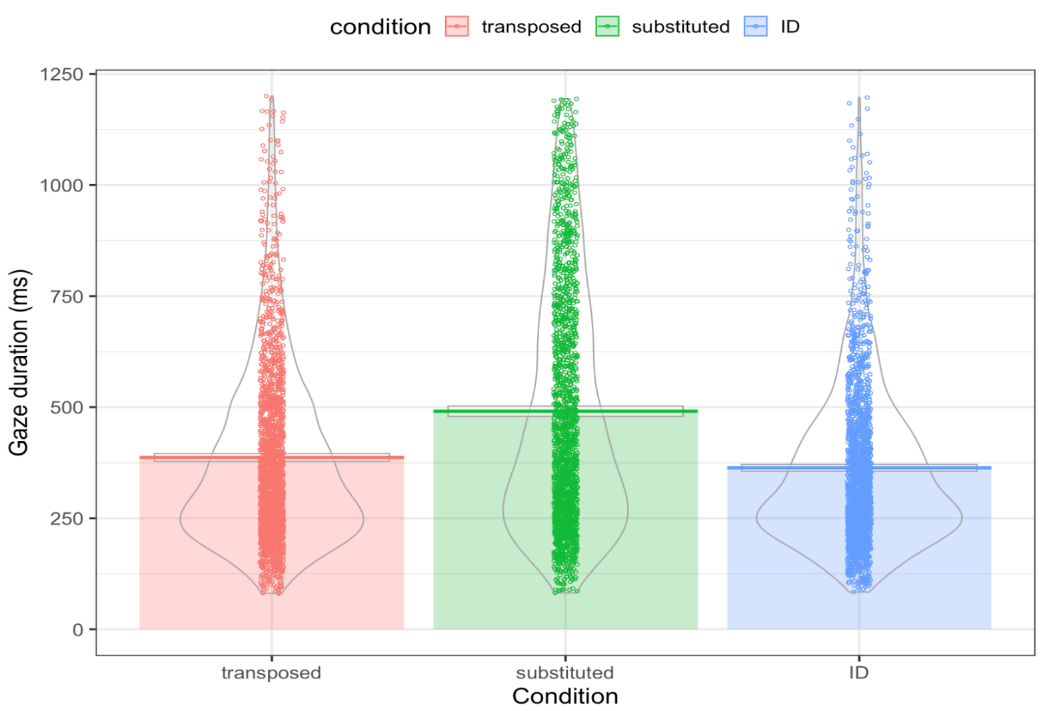
**

*Figure 6*. First fixation duration for target words in each of the three conditions: transposed, substituted and ID, where the raw data is plotted as points, the bars and horizontal line display the means, the boxes around the line indicate 95% confidence intervals assuming a normal sampling distribution, and the violins indicate the density.

*Table 9* also displays summary statistics of the LMM model with contrasts of condition (TL and SL vs ID, and TL vs SL) and proficiency, as well as the interactions between contrasts of condition and proficiency, as specified. For first fixation duration, our model fitted to log-transformed data with intercept-only structures (as determined by *buildmer*) for the random effects was: *(lmer(log(eye movement measure)~ Condition*English LexTALE + (1 | participant) + (1 | item))*.

The model fitted to log-transformed data indicated that simple effects of condition on each eye-movement measure were significant but the effect of English LexTALE was non-significant. Interestingly, there is a significant interaction between TL + SL vs ID and English LexTALE, but not between TL vs SL and English LexTALE. Figure 7 indicates that this interaction is driven by TL and SL words being more costly in first-fixation duration than for less skilled readers.

**
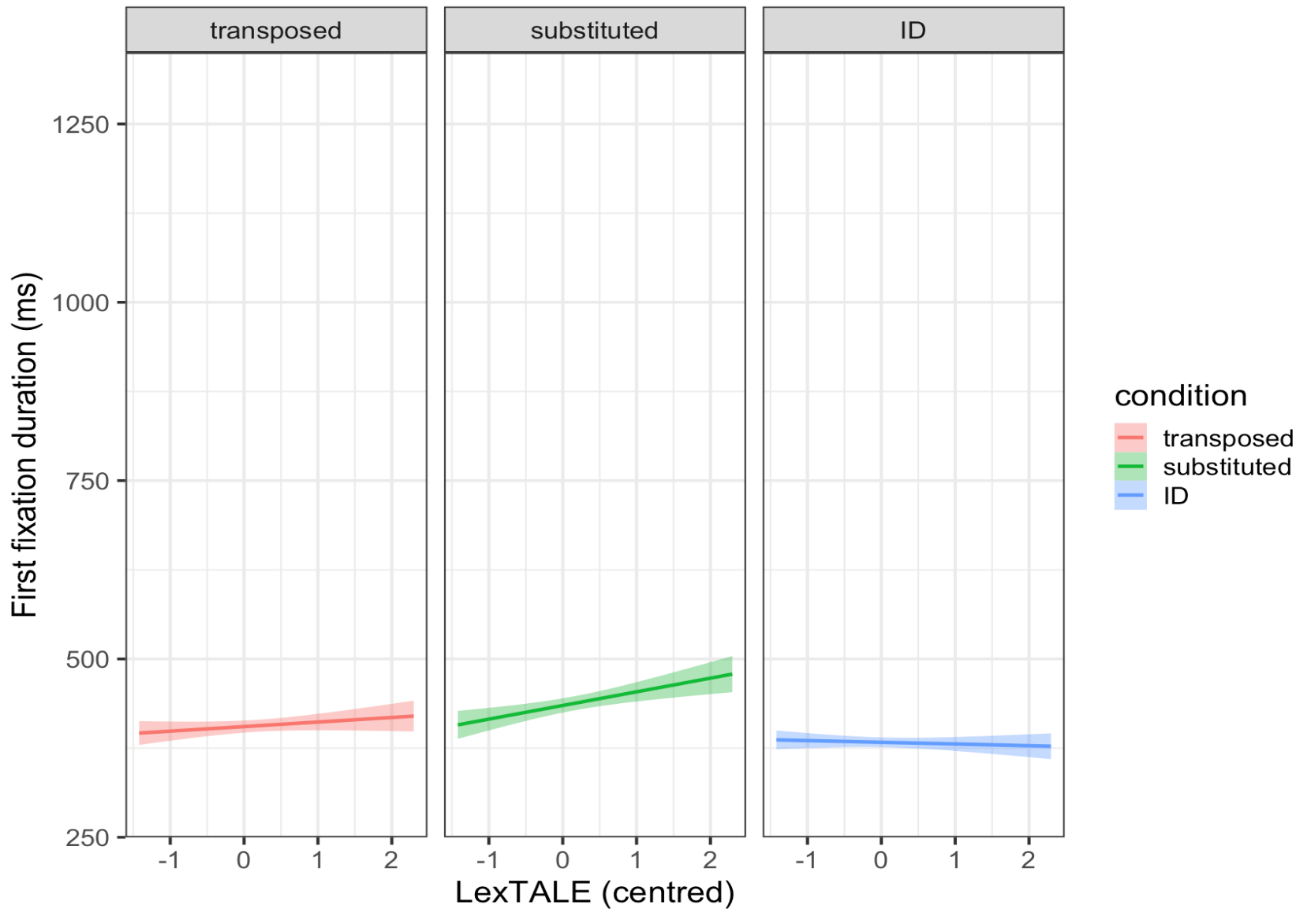
**

*Figure 7.* First fixation duration for target words in each condition (transposed, substituted and ID) plotted against the participants’ corresponding scaled and centred English LexTALE scores. Solid lines indicate the linear relationship and shaded areas indicate 95% confidence intervals.

Table 9

*Linear mixed-effect model outcomes for first fixation duration with predictors of manipulation (TL and SL vs ID) and manipulation type (TL vs SL), and a separate model with the addition of predictors English LexTALE, interaction between manipulation and English LexTALE, and the interaction between manipulation type and English LexTALE.*

| *Predictors* | **First fixation duration** | | | | | | | | | |
| --- | --- | --- | --- | --- | --- | --- | --- | --- | --- | --- |
|  | **Replication model** | | | | | **Proficiency model** | | | | |
|  | *Estimates* | *SE* | *95% CI* | *t* | *p* | *Estimates* | *SE* | *95% CI* | *t* | *p* |
| (Intercept) | 5.54 | 0.02 | 5.50 – 5.57 | 317.23 | **<0.001** | 5.54 | 0.02 | 5.50 – 5.57 | 311.92 | **<0.001** |
| Condition (TL + SL vs ID) | -0.02 | 0.00 | -0.03 – -0.01 | -6.08 | **<0.001** | -0.02 | 0.00 | -0.03 – -0.01 | -5.76 | **<0.001** |
| Condition (TL vs SL) | 0.03 | 0.01 | 0.02 – 0.04 | 4.88 | **<0.001** | 0.03 | 0.01 | 0.02 – 0.04 | 4.76 | **<0.001** |
| English LexTALE |  |  |  |  |  | 0.01 | 0.02 | -0.03 – 0.04 | 0.28 | 0.776 |
| Condition (TL + SL vs ID)*English LexTALE |  |  |  |  |  | -0.01 | 0.00 | -0.01 – -0.00 | -1.98 | **0.048** |
| Condition (TL vs SL)*English LexTALE |  |  |  |  |  | 0.00 | 0.01 | -0.01 – 0.01 | 0.47 | 0.635 |

*Note*. Significant effects are indicated in bold. SE: standard error.

**First-pass regression ratio (post-hoc)**

*Table 10* displays Generalised Linear Mixed-effects models (GLMM) summary statistics for first pass regression ratio, with contrasts of condition (TL and SL vs ID, and TL vs SL), as specified. Our model fitted to log-transformed data with intercept-only structures (as determined by *buildmer*) for the random effects was *(glmer(log(eye movement measure)~ condition + (1 | participant) + (1 | item), family= binomial)*. Our model suggested that both contrasts of condition (TL + SL vs ID and TL vs SL) were not significant predictors of first pass regression ratio. This suggests that regression rates were not impacted by any manipulations of the target word.

*Table 10* also displays summary statistics of the GLMM model with contrasts of condition (TL and SL vs ID, and TL vs SL) and proficiency, as well as the interactions between contrasts of condition and proficiency, as specified. For first pass regression ratio, our model fitted to binomial data with intercept-only structures (as determined by *buildmer*) for the random effects was: *(glmer(log(eye movement measure)~ Condition*English LexTALE + (1 | participant) + (1 | item), family= binomial)*. Our model suggested that none of the predictors were significant predictors of first pass regression ratio.

Table 9

*Generalised linear mixed-effect model outcomes for first fixation duration with predictors of manipulation (TL and SL vs ID) and manipulation type (TL vs SL), and a separate model with the addition of predictors English LexTALE, interaction between manipulation and English LexTALE, and the interaction between manipulation type and English LexTALE.*

|  | **First-Pass Regression Ratio** | | | | | **First-Pass Regression Ratio** | | | | |
| --- | --- | --- | --- | --- | --- | --- | --- | --- | --- | --- |
| *Predictors* | *Estimates* | *SE* | *95% CI* | *t* | *p* | *Estimates* | *SE* | *95% CI* | *t* | *p* |
| (Intercept) | 0.15 | 0.01 | 0.12 – 0.18 | -19.91 | **<0.001** | 0.15 | 0.01 | 0.12 – 0.18 | -19.77 | **<0.001** |
| Condition (TL + SL vs ID) | 0.96 | 0.03 | 0.91 – 1.01 | -1.57 | 0.117 | 0.96 | 0.03 | 0.91 – 1.01 | -1.64 | 0.100 |
| Condition (TL vs SL) | 1.08 | 0.05 | 0.99 – 1.18 | 1.64 | 0.102 | 1.08 | 0.05 | 0.99 – 1.18 | 1.63 | 0.103 |
| English LexTALE |  |  |  |  |  | 1.01 | 0.09 | 0.85 – 1.21 | 0.15 | 0.878 |
| Condition (TL + SL vs ID)*English LexTALE |  |  |  |  |  | 1.02 | 0.03 | 0.96 – 1.08 | 0.64 | 0.522 |
| Condition (TL vs SL)*English LexTALE |  |  |  |  |  | 1.00 | 0.05 | 0.90 – 1.09 | -0.10 | 0.920 |

*Note*. Significant effects are indicated in bold. SE: standard error.
